# Deployment and utilization of next-generation sequencing of *Plasmodium falciparum* to guide anti-malarial drug policy decisions in sub-Saharan Africa: opportunities and challenges

**DOI:** 10.1186/s12936-019-2853-4

**Published:** 2019-09-03

**Authors:** Deus S. Ishengoma, Queen Saidi, Carol H. Sibley, Cally Roper, Michael Alifrangis

**Affiliations:** 1 National Institute for Medical Research, Tanga Centre, Tanga, Tanzania; 2 Kilimanjaro Christian Medical University College, Moshi, Tanzania; 3 Department of Genome Sciences, University of Washington, Seattle, USA; 4 London School of Hygiene & Tropical Medicine, London, UK; 5 Centre for Medical Parasitology, Department of Immunology and Microbiology, University of Copenhagen, Copenhagen, Denmark; 6 Department of Infectious Diseases, Copenhagen University Hospital, Copenhagen, Denmark

**Keywords:** Next-generation sequencing, Malaria, *Plasmodium falciparum*, Drug resistance, Sub-Saharan Africa

## Abstract

Parasite resistance against anti-malarial drugs is a major threat to the ongoing malaria control and elimination strategies. This is especially true since resistance to the currently recommended artemisinins and partner drugs has been confirmed in South East Asia (SEA) and new anti-malarial compounds are not expected to be available in the near future. Spread from SEA or independent emergence of artemisinin resistance in sub-Saharan Africa (SSA) could reverse the achievements in malaria control that have been attained in the past two decades and derail the ongoing elimination strategies. The current surveillance of clinical efficacy and resistance to anti-malarial drugs is based on efficacy trials to assess the clinical performance of anti-malarials, in vivo*/*ex vivo assessment of parasite susceptibility to anti-malarials and prevalence of known molecular markers of drug resistance. Whereas clinical efficacy trials are restricted by cost and the complex logistics of patient follow-up, molecular detection of genetic mutations associated with resistance or reduced susceptibility to anti-malarials is by contrast a simple and powerful tool for early detection and monitoring of the prevalence of resistant parasites at population level. This provides needed information before clinical failure emerges, allowing policy makers to anticipate problems and respond. The various methods previously used in detection of molecular markers of drug resistance share some limitations: low-throughput, and high costs per sample and demanding infrastructure. However, recent technological advances including next-generation sequencing (NGS) methodologies promise greatly increased throughput and reduced costs, essentially providing unprecedented potential to address different research and operational questions of relevance for drug policy. This review assesses the potential role of NGS to provide comprehensive information that could guide drug policies in malaria endemic countries and looks at the foreseeable challenges facing the establishment of NGS approaches for routine surveillance of parasite resistance to anti-malarials in SSA.

## Background

The Global Technical Strategy for malaria (GTSm) 2016–2030 (https://www.who.int/malaria/publications/atoz/9789241564991/en/) was formulated by the World Health Organization (WHO) to provide a framework to guide endemic counties toward malaria elimination. It sets out targets for the coming decade which include; 90% reduction of global malaria mortality and case incidence by 2030 (compared to 2015); eliminating malaria in at least 35 countries; and preventing re-introduction of malaria in all countries that are disease-free [1]. The interventions recommended by GTSm include current vector control methods [with long-lasting insecticide-treated bed nets (LLINs) and/or insecticide-treated bed nets (ITNs), and indoor residual spraying (IRS)] and effective case management [involving prompt diagnosis with rapid diagnostic tests (RDTs) and treatment using artemisinin-based combination therapy (ACT)]. GTSm also recommends promoting and implementing malaria surveillance as a core intervention [1]. However, the impact of these interventions on malaria burden will be undermined by insufficient funding for malaria [2], weak health systems [3, 4], resistance to insecticides by *Anopheles* vectors [5] and emergence of parasites resistant to commonly used anti-malarials [6]. Drug resistance is primarily a challenge facing control of the *Plasmodium falciparum* species, and includes resistance to artemisinins [7–9] and partner drugs [10, 11], arising in South East Asia (SEA). In particular, the threat to ACT efficacy calls for sustained surveillance to ensure prompt detection of resistance emergence and containment of its spread to other endemic countries and particularly to sub-Saharan Africa (SSA), where it is still highly effective.

Thus, parasite resistance to anti-malarial drugs is a major obstacle to current initiatives for effective control and elimination of malaria. Drug pressure is the key factor driving the emergence and spread of resistant parasites. However, other factors (related to human host, mosquito vectors and the parasites) and conditions leading to malaria treatment failure may also contribute to development of resistance [12].

Assessment and/or confirmation of resistance to anti-malarial drugs is usually determined by therapeutic efficacy studies (TES) in which the clinical efficacy of different drugs is assessed. This remains the gold standard for guiding formulation of malaria treatment policies [13]. However, TES are challenging to implement on a regular basis due to costs and issues related to logistics [12]. Alternatively, ex vivo/in vitro laboratory assessment of sensitivity of *P. falciparum* field isolates to anti-malarial compounds is possible and has the advantage that parasite susceptibility to individual drugs is obtained without interference or confounding by host immunity. However, the methodology is available only where there is adequate laboratory infrastructure and skilled human resources, and furthermore it cannot assess susceptibility to drug combinations [14].

Drug susceptibility in *P. falciparum* populations is influenced by specific mutations [single nucleotide polymorphisms (SNPs)] or to a lesser extent, amplifications of certain genes leading to copy number variants (CNVs) in the *P. falciparum* genome [15, 16]. By consecutive measurement of the occurrence of these molecular markers of drug resistance within populations, effective surveillance of temporal changes and geographical spread is feasible. A strong and reliable surveillance framework which uses molecular markers could potentially inform drug policy and support timely intervention to forestall widespread treatment failure. However, until now, the operational relevance of molecular markers of resistance for drug policy has also been limited to the few areas where surveillance is adequate. Thus, without investment in large-scale routine sampling of malaria parasites across malaria endemic regions, the provision of timely, comprehensive molecular surveillance data to guide policy has been out of reach in SSA.

Over the past three decades, molecular surveillance has largely relied on traditional low throughput genotyping methods to generate data and map the status of parasite resistance to different anti-malarial drugs [17]. Due to recent technological advancement and reduction in the costs, next-generation sequencing (NGS) methods have the potential to address different research and operational questions in a timely manner. In particular, these methods could support high quality biological and epidemiological studies, including tracking resistance to anti-malarial drugs. However, the methods need to be refined and tailored to address the operational challenges, which currently limit their application in SSA. An adaptive system would generate information to guide the choice, implementation and use of effective anti-malarial drugs in SSA.

This review paper explores opportunities and challenges of using new genomic screening tools and associated bioinformatic analysis to the surveillance of parasite resistance to anti-malarials. The goal is to outline what may be required for this approach to become operational and guide drug policy in malaria endemic countries in the future.

## A brief historical perspective of the evolution of anti-malarial drug resistance

Resistance has been described for most currently available drugs, although the intensity and geographic extent of resistance is not always known. The mechanisms of resistance and *P. falciparum* genes involved have been the subject of intensive research. For chloroquine (CQ), resistance developed almost immediately in *P. falciparum* populations following its first introduction in the late 1940s. Resistance to CQ initially emerged in Cambodia, Thailand and Colombia most likely because CQ was used there both for direct treatment and indirectly (in cooking salt), and in mass drug administration [18, 19]. From SEA, CQ resistant parasites spread towards the west and reached East Africa through India in the late 1970s [20, 21]. As CQ resistance spread in Africa, mortality increased at an alarming rate, with as high as sixfold higher death rates in children under 5 in some countries such as Senegal [22].

*Plasmodium falciparum* resistance to the antifolate combination, sulfadoxine/pyrimethamine (SP) has also been widely reported. The pattern of emergence and spread of resistance to SP was similar to that of CQ; emerging in SEA and then spreading to East Africa [23]. Although resistance to SP is widespread in Africa, the intensity of resistance is higher in the east compared to West Africa, reflecting its emergence and dispersal pattern [24, 25].

Artemisinins were originally developed from natural compounds in China in the 1970s and are highly effective at killing *P. falciparum*. ACT containing an artemisinin compound and a partner drug (mainly lumefantrine, amodiaquine and recently, piperaquine) was promoted by the WHO in the 2000s for the treatment of uncomplicated falciparum malaria [26]. Artemisinin-based combinations, namely artemether–lumefantrine, artesunate–amodiaquine and dihydroartemisinin–piperaquine are currently used in all SSA countries against uncomplicated falciparum malaria [2]. In these combinations, the rapidly-eliminated artemisinin component rapidly clears most of the parasites, and the remaining parasites are killed by the longer acting partner drug. However, reports from SEA showed that parasites have evolved partial resistance to artemisinins observed as a much slower rate of parasite clearance after artemisinin exposure [7–9, 27, 28]. Moreover, resistance to key partner drugs such as mefloquine and piperaquine is now widespread [10, 11].

## Molecular markers and anti-malarial drug resistance

Molecular markers associated with reduced response to particular drugs have been identified for different anti-malarials including CQ, SP, artemisinins and partner drugs used in ACT (Table 1). These markers can serve as simple and useful tools in screening for emergence of resistance and assessing its spread.

**Table 1:** Main molecular markers associated with reduced response to different anti-malarial drugs

Anti-malarial drugs	Genes involved	Specific mutations	References
Artemisinins			
Artemisinin and its derivatives	*pfk13*	Confirmed/validated SNPs in the propeller domain^a^	[43, 44, 46, 47]
4-Amino quinolones			
Amodiaquine	*pfcrt/pfmdr1*	*pfmdr1:* 86**Y**/Y184/1246**Y**	[52, 53]
		*pfcrt* codons 72-76 (CV**IET**/**S**VMN**T)**	[52, 54]
Chloroquine	*pfcrt*/*pfmdr1*	*pfcrt* codons 72-76 (CV**IET**/**S**VMN**T)**	[15, 55, 56]
		*pfmdr1:* 86**Y**/Y184/1246**Y**	[15, 52, 53]
Mefloquine	*pfmdr1*	Increased *pfmdr1* CNV	[57, 58]
Piperaquine	Plasmepsin 2/*pfcrt*	Increased pm2 CNV	[59, 60]
		*pfcrt* codons H95**Y**, F145**I**, M343**L**, G353**L**	[61–63]
Antifolate drugs			
Pyrimethamine	*pfdhfr*	*pfdhfr: 51* ***I*** */59* ***R*** */108* ***N***	[33, 35, 42]
Sulfadoxine	*pfdhps*	*pfdhps:436* ***A*** */437* ***G*** */540* ***E*** */581* ***G***	[35, 42]
Others			
Lumefantrine	*pfmdr1/pfcrt*	*pfmdr1:N*86/184**F**/D1246 or increased CNV	[32, 53, 57, 64]
		*pfcrt* codons 72-76 (CVMN**K)**	[32, 65]

*pfk13 *, * Plasmodium falciparum* kelch 13 gene; *pfcrt *, * P. falciparum* chloroquine resistance transporter; *pfmdr1 *, * P. falciparum* multidrug resistance 1; pfdhfr,  *P. falciparum* dihydrofolate reductase; *pfdhps*, *P. falciparum* dihydropteroate synthase; CNV, copy number variants

^a^SNPs at codons F446**I**, N458**Y**, M476***I***, Y493**H**, R539**T**, I543**T,** P553L, R561H and C580**Y** have been validated as markers associated with partial resistance to artemisinins whiles others have been reported as confirmed markers [6, 45–47]

In brief, single nucleotide polymorphisms (SNPs) in the *P. falciparum* chloroquine resistance transporter-gene (*pfcrt*) cause resistance to CQ [29, 30]. Mutations and gene amplifications in *P. falciparum* multidrug resistance 1 (*pfmdr1)* gene affect susceptibility to CQ and other 4- amino quinolones (quinine and mefloquine) as well as structurally unrelated anti-malarial drugs, such as artesunate and lumefantrine [31, 32]. Antifolates, such as SP act through sequential and synergistic inhibition of two key enzymes involved with folate biosynthesis. Pyrimethamine and related compounds inhibit the step mediated by dihydrofolate reductase (DHFR) [33, 34], while sulfones and sulfonamides inhibit the step mediated by dihydropteroate synthase (DHPS) [35, 36]. Mutations in *P. falciparum pfdhfr* and *pfdhps* genes directly reduce enzyme susceptibility causing resistance to pyrimethamine and sulfadoxine, respectively [37–41]. Different combinations of mutations in these genes have been associated with varying degrees of resistance to antifolate combinations [42]. A number of single nucleotide polymorphisms (SNPs) in the *P. falciparum kelch 13* gene (*pfk13*) have been shown to confer partial resistance to artemisinins [43, 44]; and some mutations (see Table 1) have been associated with slow clearance that characterizes the partial parasite resistance to artemisinins in SEA [6, 45–47].

These very specific genetic changes are defined by their association of a specific parasite genotype with response to a particular drug in the laboratory. These associations suggest that the prevalence of a certain genotype among the parasites in a location may be a useful surrogate to predict the efficacy of the drug to cure malaria patients who carry those parasites. This expectation was robustly supported in early studies of parasites that carried a particular combination of mutations in *pfdhfr* and *pfdhps* [42]. However, other studies have not always observed such associations, most likely because of confounding factors, such as acquired immunity [48–50].

The predictive relevance of the molecular markers to the clinical outcome of anti-malarial treatment has been difficult to establish for the artemisinins and their partner drugs. That is partly because the parasite resistance to the drug may result only indirectly from the associated genetic change in the parasite. Even more important, the response of a malaria patient to drug treatment is strongly dependent on other confounding factors such as acquired immunity mentioned above but as well, parasite biomass, pharmacokinetics, and patient compliance to the treatment protocol. These factors also have a major effect to the treatment outcome apart from the intrinsic parasite resistance [10, 51].

## Impact of molecular markers on drug policy

Although molecular markers of resistance to CQ, and sulfadoxine–pyrimethamine were discovered and used in mapping of resistance to these drugs (see for instance https://www.drugresistancemaps.org and https://www.wwarn.org/tracking-resistance), they have been of limited operational value because these markers were described only after resistance was already widespread. There are just two examples of molecular surveillance being incorporated into WHO recommendations for national policy guidelines. Both are related to the WHO recommendations for SP to be used as prophylactic intermittent preventive treatment, firstly in infants (IPTi) [66] and secondly in pregnant women (IPTp) [67]. The data are most clear for IPTp. In 2012, administration of SP was recommended for all women in the second and third trimesters of pregnancy because it was demonstrated that babies born to women who receive this preventative treatment had significantly higher birth weight and better survival during the neonatal period [68, 69]. However, these early studies were carried out at a time when SP treatment of malaria disease was still efficacious and the prevalence of the markers of SP resistance in parasite populations was correspondingly low in most regions [69]. As the prevalence of the resistance markers rose, this signaled that SP efficacy was falling and soon fell below the standard for adequate clinical treatment; SP was no longer recommended for treatment of uncomplicated malaria.

However, further studies on SP-IPTp showed that it was still beneficial as prophylactic during pregnancy despite high levels of SP resistance, as measured by prevalence of parasites carrying both triple mutant *pfdhfr* and double mutant *pfdhps* alleles analysed in a wide range of locations [70]. The policy question then became “at what prevalence of SP resistance is even the preventative effect of SP gone?” There was a clear regional difference between West Africa where triple mutant *pfdhfr* and single mutant *pfdhps* parasites were most common and IPTp remained protective. But protection was less clear in East Africa where parasites carrying both triple mutant *pfdhfr* and double mutant *pfdhp*s (often called the triple-double) were in certain limited areas accompanied by an additional *pfdhps* mutation 581G [70]. Studies conducted in North-eastern Tanzania, in an area with high prevalence of *pfdhps* 581G mutation, reported that IPTp was associated with an increased proportion of infections carrying *pfdhp*s 581G mutations, increased level of parasitaemia, and more intense placental inflammation [71]. Another study later showed that women infected with highly resistant parasites (with triple mutations at *pfdhp*s) had babies with low birthweight as compared to women infected with less resistant parasites. However, the impact of such infections with triple *pfdhp*s mutants remained inconclusive as observations have been based on limited sample sizes and IPTp with SP has continued to be used in the same and other areas with highly resistant parasites.

After considerable discussion, a WHO committee recommended that the prevalence of the triple double parasites in a site should be used to define a threshold above which SP IPTp should no longer be recommended for IPTp, as it would have minimal protective value when marker prevalence indicated resistance was too high [67]. This history demonstrated the potential value of molecular markers of SP resistance and how it might be used to guide clinical recommendation.

After identification of the *pfk13*-propeller locus as a marker of partial resistance to artemisinins, many molecular studies have been done in Africa. Reports of low prevalence of many different mutations in *pfk13* have been published from many endemic countries but in general, little evidence of slow parasite clearance has been gathered [6, 45–47]. In this important case, molecular surveillance has the potential to provide policy makers with a forecast of impending problems, rather than confirmation of an already existing one [71]. The molecular approach is especially valuable, since ACT is very widely used, so drugs are used in combination. For example, molecular surveillance can suggest that a partner drug is losing efficacy even when it is still apparently clinically effective when used in combination with an artemisinin. Conversely, recent reports show increasing prevalence of plasmepsin copy numbers (which is a marker of resistance to piperaquine in SEA) despite recent introduction of piperaquine in Africa [72].

Overall, surveillance of molecular markers of resistance to drugs currently in use have the operational potential to inform drug policy makers on the status of drug resistance at local, national and regional level. If done proactively, surveillance of molecular markers can provide advanced warning of increased prevalence of parasite resistant to drugs in use in a region. With this information, necessary changes in policy can be put in place to limit malaria attributed morbidity caused by failing drugs.

## Opportunities, challenges and priorities for application of next generation sequencing (NGS) in drug resistance surveillance

In order to provide policy-makers with annotated and timely molecular data of relevance, several prerequisites and processes have to be established and molecular analyses of drug resistance will play a central role. Different methods for detection of drug resistance markers have been developed in the last three decades and most of them have been established in various laboratories in SSA. These methods are all based on PCR, followed by various methodologies to identify the relevant SNPs or copy numbers (including PCR-RFLP, PCR-SSOP-ELISA, real-time PCR, LAMP and custom DNA micro-arrays) and a comparative assessment of these techniques was recently presented [17]. The major limitation of most of the current methods is low throughput, despite the short turn-round time. However, in recent years application of genetics and genomics methods in public health have significantly grown because of various innovations and the declining costs of individual assays.

New methods, such as targeted NGS (TNGS) and associated bioinformatics tools have recently provided possibilities for application to surveillance of anti-malarial resistance [73–76]. These methods are evolving rapidly, and the methods based on TNGS have the potential, to lower costs by allowing simultaneous assessment of large sample sets, using the capacity for automated high-throughput, high sensitivity and scalability for use in national/regional reference and research laboratories [17]. One particular advantage is that TNGS-based methods allow for pooled sequencing of many individual patient isolates, retaining the capacity to still identify the prevalence of molecular components in each original sample. Pooling of samples can be done at different stages either before or after DNA extraction and this possibility can significantly reduce the costs and make it logistically possible to analyse rapidly a large number of samples [77]. This expansion could support far wider and deeper surveillance of the temporal and spatial distribution of molecular markers, closing the wide gaps in the overall maps currently available.

Despite the potential, the initial investments in infrastructure for NGS are high, and TNGS-based methods require highly skilled personnel to perform sample processing and the necessary bioinformatics data analysis (Bailey et al. pers.commun.). Currently, these pose significant barriers in most of SSA. Thus, opportunities for establishing and applying TNGS for surveillance of drug resistance in SSA is lagging behind due to these (and additional challenges, see below), that must be urgently tackled.

### Appropriate choice of sample collection sites for surveillance of drug resistance markers

One major obstacle to efficient, timely surveillance of drug resistance markers is the methodological approach for appropriate sample collection. Previous studies have largely relied on opportunistic samples from sites of interest and/or convenience. As a result, the maps of malaria-relevant parameters show geographically and temporally sporadic distribution with some regions/sites contributing to significant molecular knowledge but others are not represented at all [25]. This disparity is increasingly common as malaria transmission decreases overall. At this point a majority of malaria-infected individuals live in rural areas and hard-to reach malaria transmission hotspots, away from centres of populations with laboratory infrastructure, reliable electricity and transport infrastructure.

Currently, there are no guidelines for selection of surveillance sites to potentially cover areas with high risk of small populations particularly at risk for selection of resistant parasites. Perhaps worse, sites vulnerable to importation of highly multi drug resistant parasites from SEA where artemisinin resistance is currently confined may not be monitored at all. Even in countries with ongoing TES and molecular surveillance such as Tanzania [78, 79], the current surveillance sites might not be suitable for maximizing a chance of detecting emerging artemisinin or partner drug resistance. Guidelines for selection of the sites with sufficient geographical coverage and international connectivity are required to provide a standardized framework for inclusion of high-risk areas in order to facilitate detection of both local and imported resistant parasites.

New initiatives should be made to leverage on regular programmes and platforms which are currently implemented to obtain samples which will greatly enhance the capacity to generate nationally representative molecular data. Such programmes include national-wide demographic and health surveys (DHS), malaria indicator surveys (MIS), school-based malaria parasitological surveys and testing of pregnant women at the first antenatal visits. Materials collected during these surveys can be cost free (RDTs) or relatively cheap to add on such as dried blood spots on filter papers (DBS). This will potentially overcome the limited sampling of convenience sampling of a few TES sites to provide population representative sampling.

### Types of sample collection of *Plasmodium falciparum* positives for drug resistance surveillance

Parasite samples for molecular surveillance of drug resistance are usually obtained by collecting small amount of blood samples, dried on filter paper (DBS). This has the advantage of being relatively non-invasive, but it is still dependent on a well-designed sampling protocol for collection, preservation and record keeping as part of malaria epidemiological or clinical trial studies. However, malaria RDTs have also been shown to be a good source of parasite DNA [80, 81], so retention of positive RDTs from patients represents a particularly attractive alternative. In particular, these require no extra steps for the patient and staff and the discarded RDTs can be stored easily at health centres/sentinel sites on a routine basis. Thus, these assessments of discarded RDTs would be a cost-effective strategy to facilitate creation of a sample repository for molecular surveillance of different markers of drug resistance; and this approach has been pilot tested in Senegal [82]. Such setup would support an environmentally safe disposal of used RDTs that would otherwise be thrown away.

### Advocacy for investments in molecular analytical expertise in SSA

Currently, there is as mentioned, a lack of local capacity for genomic studies in most of SSA countries due to poor laboratory infrastructure, the shortage of skilled researchers and technicians, and lack of computing facilities. Despite a recent increase in funding from international funders, there is a lack of/inadequate support by African governments and international donors. African scientists and their collaborators need to advocate for increased domestic funding to complement the current support by international agents to build and sustain local capacity including human resources and laboratory facilities. This will increase the capacity of African institutions to attract, train and retain skilled personnel with expertise in genomics and bioinformatics; and effectively adopt and utilize genomic methods such as TNGS to support malaria elimination in Africa.

However, in most of SSA countries, there has also been limited engagement of policy makers and no calls for application of genetic/genomic studies for addressing different epidemiological questions with operational relevance, such as monitoring of drug resistance. As result, governments in these countries are unable to fund genomic studies partly due to neglect and insufficient funds within the domestic budget. Most of African government are unable to meet the financial demands required for implementing malaria control. Because of this, funding molecular surveillance of anti-malarial resistance may be seen as a low priority compared to supporting malaria interventions such as bed nets, RDTs and anti-malarial drugs. There is an urgent need to increase awareness among policy makers of the potential application of molecular surveillance for tracking anti-malarial drug resistance, particularly in the light of developments in NGS tools that potentially could facilitate timely production of informative molecular data.

To increase acceptability and utilization of molecular surveillance of drug resistance, it is critical to involve NMCPs, local partners such as academic and research institutions, and the respective local governments/communities. National programmes must attain leadership and ownership of the initiatives to ensure their sustainability. Training of key staff and some members the programme management is essential to give them a better understanding of molecular surveillance and use of genomics data to address the challenges and limitations to effectiveness of the current interventions. NMCP, local government authorities and communities also need to be sensitized and equipped to appreciate how genetic data can potentially support and influence the process of changing malaria treatment policies. These national and local stakeholders should be involved in the planning, and implementation of molecular surveillance activities. The studies should be designed and implemented by NMCPs in collaboration with their partners, to specifically address questions of relevance and priority to national and local contexts. There should be a strong partnership between researchers and NMCP and local authorities with the required skills to share and appropriately disseminate research findings to key stakeholders and policy makers. Innovative approaches, such as maps and interactive visualization tools, need to be developed and the findings need to be reported in a language that is clearly understood by the target audience. A strong and well-designed partnership between NMCP and other local and international partners is critical to ensure that molecular surveillance data and findings of anti-malarial drug resistance studies are appropriately utilized to guide policy formulation at national and global levels.

### Procurement of reagents and consumables for NGS in SSA

In most of the countries in SSA, there is poor/lack of a reliable supply chain for reagents and consumables to facilitate timely procurement and delivery of the materials. Although research materials are ordered through the collaborating laboratories in Europe and USA, they often get stuck at the customs for several months before they can be delivered to the laboratories. There is a growing market of local suppliers of reagents and consumables, which is highly welcomed. However, their supplies are over-priced and not always of similar high quality as compared to supplies obtained by laboratories outside SSA. Improvement in these areas is possible but that will depend on the level of collaboration between researchers and different stakeholders at national, regional/province and district levels.

## Conclusion

NGS-based methods offer enormous potential to generate extensive, high quality molecular data to support tracking the emergence and spread of drug resistant parasites. If these systems could be developed, they could provide useful information to guide policy-makers on malaria treatment policies in close to real-time. However, several challenges need to be resolved to enable malaria endemic countries in SSA to fully utilize genomics and bioinformatics tools in the ongoing malaria control/elimination strategies and guiding anti-malarial treatment policies. Training a critical mass of SSA researchers with expertise to generate and interpret NGS data and increasing the number of NGS platforms in SSA is essential. Then, to decide on a sampling strategy that will provide routine and temporal molecular data from across SSA is necessary to secure timely data on molecular markers and enable early warning of any signs of resistance to ACT on the continent.

Appropriate initiatives will be required to engage NMCPs and help them to appreciate the value that molecular surveillance can bring, in addressing operational issues relevant to their local responsibilities. Researchers from malaria endemic countries especially in SSA should take a leading role in creating the awareness of key stakeholders and increased funding particularly from their governments and international agencies. They should also advocate for national/regional reference laboratories, which will build the capacity to locally generate genomics data to support malaria elimination in their respective countries.

## Abbreviations

ACT: artemisinin-based combination therapy
AL: artemether–lumefantrine
ASAQ: artesunate–amodiaquine
CNVs: copy number variants
CQ: chloroquine
DBS: dried blood spots
DNA: deoxyribonucleic acid
DP: dihydroartemisinin–piperaquine
GTSm: Global Technical Strategy for malaria
IPTi: intermittent preventive treatment in infants
IPTp: intermittent preventive treatment in pregnancy
IRS: indoor residual spraying
ITNs: insecticide treated bed nets
LLINs: long-lasting insecticide-treated bed nets
RDTs: rapid diagnostic tests
NGS: next-generation sequencing
*pfcrt*: *P. falciparum* chloroquine resistance transporter gene
*pfdhfr*: *P. falciparum* dihydrofolate reductase
*pfdhps*: *P. falciparum* dihydropteroate synthase
*pfk13*: *P. falciparum kelch 13* gene
*pfmdr1*: *P. falciparum* multidrug resistance 1 gene
SEA: South East Asia
SNPs: single nucleotide polymorphisms
SP: sulfadoxine–pyrimethamine
SSA: sub-Saharan Africa
TES: therapeutic efficacy studies
TNGS: targeted NGS
WHO: World Health Organization
